# Supplementary material for: The Bacterial Urban Resistome: Recent Advances
Source: Antibiotics (Basel). 2022 Apr 12;11(4):512. doi: 10.3390/antibiotics11040512 (PMC9030810; doi:10.3390/antibiotics11040512)
Supplement: Supplementary file 1 [file antibiotics-11-00512-s001.zip › antibiotics-1673646-supplementary.pdf]

Figure 3 has been produced by using the following methodology. The search has been completed in PubMed.

1) Filter used to search Hospitals:

("hospita*"[All Fields] AND "Ab GENE"[All Fields] AND (("anti bacterial agents"[Pharmacological Action] OR "anti bacterial agents"[MeSH Terms] OR ("anti bacterial"[All Fields] AND "agents"[All Fields]) OR "anti bacterial agents"[All Fields] OR "antibiotic"[All Fields] OR "antibiotics"[All Fields] OR "antibiotic s"[All Fields] OR "antibiotical"[All Fields]) AND "resistanc*"[All Fields])) AND ((ffrft[Filter]) AND (fft[Filter]) AND (1990:3000/12/12[pdat]))

antibiotic: "anti-bacterial agents"[Pharmacological Action] OR "anti-bacterial agents"[MeSH Terms] OR ("anti-bacterial"[All Fields] AND "agents"[All Fields]) OR "anti-bacterial agents"[All Fields] OR "antibiotic"[All Fields] OR "antibiotics"[All Fields] OR "antibiotic's"[All Fields] OR "antibiotical"[All Fields]

2) Filter used to search Pets or in alternative "companion anima*"

("pet"[All Fields] AND "Ab GENE"[All Fields] AND (("anti bacterial agents"[Pharmacological Action] OR "anti bacterial agents"[MeSH Terms] OR ("anti bacterial"[All Fields] AND "agents"[All Fields]) OR "anti bacterial agents"[All Fields] OR "antibiotic"[All Fields] OR "antibiotics"[All Fields] OR "antibiotic s"[All Fields] OR "antibiotical"[All Fields]) AND "resistanc*"[All Fields])) AND ((ffrft[Filter]) AND (fft[Filter]) AND (1990:3000/12/12[pdat]))

antibiotic: "anti-bacterial agents"[Pharmacological Action] OR "anti-bacterial agents"[MeSH Terms] OR ("anti-bacterial"[All Fields] AND "agents"[All Fields]) OR "anti-bacterial agents"[All Fields] OR "antibiotic"[All Fields] OR "antibiotics"[All Fields] OR "antibiotic's"[All Fields] OR "antibiotical"[All Fields]

Note plasmid pET was excluded.

3) Filter used to search recreational and park
(("recreational"[All Fields] OR "park*"[All Fields]) AND (("anti bacterial agents"[Pharmacological Action] OR "anti bacterial agents"[MeSH Terms] OR ("anti bacterial"[All Fields] AND "agents"[All Fields]) OR "anti bacterial agents"[All Fields] OR "antibiotic"[All Fields] OR "antibiotics"[All Fields] OR "antibiotic s"[All Fields] OR "antibiotical"[All Fields]) AND "resistanc*"[All Fields]) AND "tetM"[All Fields]) AND ((ffrft[Filter]) AND (fft[Filter]) AND (1990:3000/12/12[pdat]))

antibiotic: "anti-bacterial agents"[Pharmacological Action] OR "anti-bacterial agents"[MeSH Terms] OR ("anti-bacterial"[All Fields] AND "agents"[All Fields]) OR "anti-bacterial agents"[All Fields] OR "antibiotic"[All Fields] OR "antibiotics"[All Fields] OR "antibiotic's"[All Fields] OR "antibiotical"[All Fields]

4) Filter used to search sewage

("sewage"[All Fields] AND (("anti bacterial agents"[Pharmacological Action] OR "anti bacterial agents"[MeSH Terms] OR ("anti bacterial"[All Fields] AND "agents"[All Fields]) OR "anti bacterial agents"[All Fields] OR "antibiotic"[All Fields] OR "antibiotics"[All Fields] OR "antibiotic s"[All Fields] OR "antibiotical"[All Fields]) AND "resistanc*"[All Fields]) AND "tetA"[All Fields]) AND ((ffrft[Filter]) AND (fft[Filter]) AND (1990:3000/12/12[pdat]))

antibiotic: "anti-bacterial agents"[Pharmacological Action] OR "anti-bacterial agents"[MeSH Terms] OR ("anti-bacterial"[All Fields] AND "agents"[All Fields]) OR "anti-bacterial agents"[All Fields] OR "antibiotic"[All Fields] OR "antibiotics"[All Fields] OR "antibiotic's"[All Fields] OR "antibiotical"[All Fields]
